# COVID-19: not a contraindication for surgery in patients with proximal femur fragility fractures

**DOI:** 10.1186/s13018-020-01800-9

**Published:** 2020-07-28

**Authors:** Ilaria Morelli, Francesco Luceri, Riccardo Giorgino, Riccardo Accetta, Paolo Perazzo, Laura Mangiavini, Nicola Maffulli, Giuseppe M. Peretti

**Affiliations:** 1 Residency Program in Orthopaedic and Traumatology, University of Milan, Via Festa del Perdono 7, 20122 Milan, Italy; 2 Equipe of Regenerative and Reconstructive Orthopaedics (EUORR) Unit, IRCCS Istituto Ortopedico Galeazzi, Via Riccardo Galeazzi 4, 20161 Milano, Italy; 3 Trauma Unit and Emergency Department, IRCCS Istituto Ortopedico Galeazzi, Via Riccardo Galeazzi 4, 20161 Milano, Italy; 4 Anesthesiology Service and Intensive Care Unit, IRCCS Istituto Ortopedico Galeazzi, Via Riccardo Galeazzi 4, 20161 Milano, Italy; 5 Department of Biomedical Sciences for Health, University of Milan, Via Mangiagalli 31, 20133 Milan, Italy; 6 Department of Medicine, Surgery and Dentistry, University of Salerno, Via S. Allende, 84081 Baronissi, (SA) Italy; 7 Barts and the London School of Medicine and Dentistry, Centre for Sports and Exercise Medicine, Mile End Hospital, Queen Mary University of London, 275 Bancroft Road, London, E1 4DG UK; 8 School of Pharmacy and Bioengineering, Keele University School of Medicine, Thornburrow Drive, Stoke on Trent, ST4 7QB UK

**Keywords:** COVID-19, Proximal femur fractures, Prognosis, Surgical treatment, Fragility fractures

During the SARS-CoV-2 outbreak, Italy was the leading country for the number of new positive cases from March 8 2020, to March 21, and the first for the total number of deaths from March 19, to April 12 2020 [1–4]. The Italian National Healthcare System required the conversion of many wards into COVID-19 care units, and the suspension of deferrable treatments, surgical procedures and outpatient visits, to dedicate human and material resources to free Intensive Care Unit (ICU) beds. In the most struck regions, some hospitals were designated as hubs for non-delayable treatments [5, 6]. Our institution (IRCCS Orthopaedic Institute Galeazzi) was chosen amongst the Trauma Hubs.

Orthopaedic trauma surgery focused mainly on femoral fragility fractures in the elderly, since the “lockdown” began on March 10, 2020. Proximal femur fractures carry a high mortality rate [7], and the standard of care involves surgery within 48 h from the trauma [8]. These elderly patients are also the most susceptible to the nefarious consequences of COVID-19 [9]. Thus, orthopaedic surgeons face the daily dilemma of performing life-saving surgery on patients who, given their severe respiratory compromise, have a higher risk of peri-operative death. Preliminary reports from Wuhan, Bergamo (the Italian province with the highest number of cases) and Spain drew different conclusions on the possible benefit effects of surgery on COVID-19 patients with proximal femur fractures [10–12]. In the first case series, six patients reported proximal femur fractures, but only three were considered eligible for surgery because of no signs of pneumonia at CT scan or non-severe respiratory symptoms [10]. Three of them died, two after conservative management. Of the three surviving patients, two underwent surgery: the authors concluded that patients with proximal femoral fractures and COVID-19 have a higher risk of death. In a series of 16 such patients in Bergamo (Lombardy), Italy [11], three patients died before surgeries for respiratory failure. The other 13 patients (temperature < 38 °C, pO_2_ > 90% and no signs of multiorgan dysfunction) underwent surgery, with four dying in the first postoperative week. Oxygen saturation improved after surgery in the survivors. Finally, a multicentre observational study on 136 proximal femoral fractures reported an overall 30.4% mortality in COVID-19 patients. The mortality rate was 67% in COVID-19 patients treated non-operatively, and only 4% in patients who underwent surgery [12].

The early outcomes (March 17–April 17, 2020) from our Trauma hub endorse the “to fix” faction. Ten swab-confirmed COVID-19 patients (8 women and 2 men) with a mean age of 83.9 ± 7.4 years (range 72-98) underwent surgical treatment for their proximal femoral fractures within 48 h from admission at our facility: 8 patients received intramedullary nailing for AO 31A fractures, and 2 received hemiarthroplasty for AO 31B fractures [13]. The mean oxygen saturation on admission was 94.4% (range 90-97), breathing ambient air by 9 patients and 4 litres/minute oxygen through Venturi mask by 1 patient. In 6 patients, atypical pneumonia was evident on plain chest radiographs on admission. Three patients underwent a further plain chest radiograph after surgery (one showed improvement, one a stable pulmonary involvement and in one patient the pneumonia worsened). Four patients developed systemic complications, including respiratory failure as reported in Table 1. Only two of these patients died, both 8 days after surgery: one of them had pneumonia on plain chest radiographs on admission. Both these patients presented the lowest oxygen saturation breathing ambient air on admission (93% and 90%, respectively). The mean length of stay in the Trauma Unit was 12.9 ± 6.9 days (range 7-29), and the surviving patients were finally discharged to our in-hospital rehabilitation unit with an improved or stable oxygen saturation, with 7 of the 8 surviving patients needing no further oxygen support.

**Table 1:** Patients’ demographics, comorbidities, surgical and clinical details, respiratory parameters and need of oxygen support

	Case n°	1	2	3	4	5	6	7	8	9	10
	**Sex**	F	F	M	F	F	F	F	F	F	M
	**Age**	85	72	85	89	77	98	81	81	80	91
	**Hospital stay (days)**	22	8	13	9	9	9	12	12	29	7
	**Clinical history**	AF, PD, NIDDM			HTN, HT	HTN, PD, HCL, MPD	Coronary artery disease	Coronary artery disease	HTN	HTN, PD	
	**Fracture**	Left, AO31A	Left, AO31A	Left, AO31A	Right, AO31A	Right, AO31A	Right, AO31A	Right, AO31A	Left, AO31A	Right, AO31B	Right, AO31B
	**SARS-CoV-2 quantitative RT-PCR**	Positive	Positive	Positive	Positive	Positive	Positive	Positive	Positive	Positive	Positive
	**Surgery**	PFN	PFN	PFN	PFN	PFN	PFN	PFN	PFN	HA	HA
	**Postoperative blood transfusions (N)**	0	0	0	0	2	1	0	2	2	1
	**Complications**	-	-	UTI, Pericardial effusion	Respiratory failure	-	Respiratory failure	-	-	UTI	Respiratory failure
	**Chest X-ray**	Positive	Positive	Negative	Positive	Positive	Negative	Positive	Negative	Positive	Negative
	**Mechanical ventilation**	No	No	No	No	No	No	No	No	No	No
**Oxygen support through Venturi mask (L/min)**	**Admission**	No (SaO2 94% in AA)	No (SaO2 96% in AA)	No (SaO2 95% in AA)	No (SaO2 93% in AA)	4 L/min (SaO2 94%)	No (SaO2 90% in AA)	No (SaO2 97% in AA)	No (SaO2 96% in AA)	No (SaO2 93% in AA)	No (SaO2 96% in AA)
	**First 3 days of hospitalization**	6 L/min	8 L/min	6 L/min	No	4 L/min	2 L/min	2 L/min	2 L/min	4 L/min	8 L/min
	**Major level needed during hospitalization**	10 L/min	8 L/min	6 L/min	No	6 L/min	4	2	2	8	8
	**Discharge**	No (SaO2 96% in AA)	No (SaO2 96% in AA)	No (SaO2 96% in AA)	-	No (SaO2 96% in AA)	-	2 L/min (SaO2 96%)	No (SaO2 96% in AA)	No (SaO2 93% in AA)	No (SaO2 96% in AA)
	**Discharge mode**	COVID-19 Rehabilitation Unit	COVID-19 Rehabilitation Unit	COVID-19 Rehabilitation Unit	Death	COVID-19 Rehabilitation Unit	Death	COVID-19 Rehabilitation Unit	COVID-19 Rehabilitation Unit	COVID-19 Rehabilitation Unit	COVID-19 Rehabilitation Unit
	**Follow-up (days)**	31	29	23	18	18	15	14	16	39	35

Abbreviations: *PFN* proximal femoral nailing; *HA* hemiarthroplasty; *RT-PCR* real time polymerase chain reaction; *AF* atrial fibrillation; *HTN* hypertension; *HT* hypothyroidism; *HCL* hypercholesterolemia; *NIDDM* diabetes mellitus; *PD* Parkinson’s disease; *MPD* myeloproliferative disorder; *UTI* urinary tract infection; *AA* ambient air

Our patients exhibited the lowest death rate (20%) to date, but, given the heterogeneous data reported by the other two case series, and the missing individual patient data of the multicentric study, the reasons for this favourable finding are unclear. In our centre, surgery for proximal femur fractures in the elderly is performed within 48 h from admission in more than 95% of patients, and this was the case for all COVID-19 patients. Two of the three operated patients reported by Mi et al. received surgery 5, 2 and 3 days after admission [10]. Catellani et al. reported surgery within 24 h after admission for 10 patients, whilst in three cases, it was postponed for haemorrhagic risk as the patients were using anticoagulant drugs [11]. Muñoz-Vives reported a mean delay from admission to surgery of 2.4 days (range 0-13) [12]. Secondly, an internal protocol, based on the previously published evidence of microvascular pulmonary thrombosis in patients with COVID-19, established the administration of low molecular weight heparin, doubling the prophylactic dose (enoxaparin sodium 4000 U.I. twice daily), as the nasopharyngeal swab showed positivity for SARS-CoV-2 [14]. The thromboembolic prophylactic therapy, if administered, was not reported by Mi and Muñoz-Vives, whilst Catellani et al. do not specify the dosage they used [10–12]. Finally, the presence of different SARS-CoV-2 clusters may explain the differences with the Chinese and Spanish studies, but it is unlikely that the viral type differed between Milan and Bergamo, two cities 60 km apart [15].

Furthermore, we retrospectively analysed the evolution of their laboratory parameters at admission, 1st and 5th ± 2 postoperative days (POD1 and POD 5 ± 2) performing a univariate repeated measures ANOVA with Greenhouse-Geisser correction and post hoc tests using the Bonferroni adjustment (statistical significance set at *p* ≤ 0.05; *F* statistic reported as *F*(degrees of freedom_time_, degrees of freedom_error_) = *F* value, *p* value). Single-patients laboratory parameters are available online as Additional file 1. Our patients demonstrated a significant reduction in leukocyte and neutrophils count and an increase in lymphocyte relative count (Table 2) comparing POD1 and POD 5 ± 2, possibly explained by the time from surgery and by the COVID-19 infection resolution, with an improvement of the peculiar SARS-CoV-2-related lymphopenia [16]. This is a noteworthy finding, considering that a low lymphocyte percentage has been considered a negative prognostic factor for COVID-19 [17].

**Table 2:** Comparison of patients’ mean laboratory parameters at admission and postoperative days 1 and 5 ± 2

Laboratory parameters	Mean value ± SD at D0	Mean value ± SD at POD 1	Mean value ± SD at POD 5 ± 2	Post hoc test (***p*** value)	***F*** statistic (ANOVA)
**Leucocyte count (× 10**^**3**^**/mL)**	10.42 ± 3.4	10.46 ± 2.8	8.65 ± 3.1	D0 vs POD1: *p* = 1 POD1 vs 5 ± 2: *p* = 0.045	*F*_(1.585, 14.261)_ = 5.644, *p* = 0.02
**Absolute neutrophil count (× 10**^**3**^**/mL)**	8.46 ± 2.9	8.46 ± 2.4	6.49 ± 2.6	D0 vs POD1: *p* = 1 POD1 vs 5 ± 2: *p* = 0.026	*F*_(1.849, 16.645)_ = 6.733, *p* = 0.008
**Relative lymphocyte count (%)**	11.4 ± 3.2	11.2 ± 5.9	16.3 ± 6.1	D0 vs POD1: *p* = 1 POD1 vs 5 ± 2: *p* = 0.02	*F*_(1.602, 14.420)_ = 5.611, *p* = 0.02

Univariate repeated measures ANOVA with Greenhouse-Geisser correction and post hoc tests using the Bonferroni adjustment (statistical significance set at *p* ≤ 0.05)

*F* statistic reported as *F*(degrees of freedom_time_, degrees of freedom_error_) = *F* value, *p* value)

Only significant outcomes are reported in this table

Abbreviations: *D0* admission day, *SD* standard deviation, *POD* postoperative day

The beneficial effects of early standard surgical care for proximal femoral fractures in the elderly seem to be confirmed also in COVID-19 patients. In-hospital management of fracture-related and cytokine-induced musculoskeletal pain in COVID-19 patients should take into account the recent warnings about the use of common NSAIDs in case of SARS-CoV-2 infection [18–20]. Nevertheless, the mortality after proximal femur fractures remains high, especially during the first year after the fracture, and COVID-19 needs a very long time for viral clearance [21, 22]. The contribution of surgery to improve respiratory and laboratory values in COVID-19 patients with femur fractures should be verified by further studies with a longer follow-up and with control groups.

## Supplementary information

**Additional file 1.** Laboratory values during hospitalization.

## Data Availability

All data generated or analysed during this study are included in this published article and its supplementary information files.
