# Supplementary material for: COVID-19: not a contraindication for surgery in patients with proximal femur fragility fractures
Source: J Orthop Surg Res. 2020 Jul 28;15:285. doi: 10.1186/s13018-020-01800-9 (PMC7385330; doi:10.1186/s13018-020-01800-9)
Supplement: Supplementary file 1 — Additional file 1. Laboratory values during hospitalization. [file 13018_2020_1800_MOESM1_ESM.docx]

**Additional file 1: Laboratory values during hospitalization.**

|  | **Case n°** | **1** | **2** | **3** | **4** | **5** | **6** | **7** | **8** | **9** | **10** |
| --- | --- | --- | --- | --- | --- | --- | --- | --- | --- | --- | --- |
| **Pre-operative** | **Haematocrit (%)**  NVF 34.1-44.9; NVM 40.1-51.0 | 35.8 | 27.8 | 42.2 | 45 | 29.5 | 28.9 | 30.1 | 26.8 | 39.4 | 30.5 |
|  | **Haemoglobin (g/dL)**  NVF 11.2-15.7; NVM 13.7-17.5 | 11.9 | 8.9 | 13.9 | 14.3 | 10.1 | 9 | 9.5 | 8.8 | 12.5 | 9.4 |
|  | **White blood-cell count (10^3/μL)**  NVF 3.98-10.04; NVM 4.23-9.07 | 8.12 | 12.12 | 8.42 | 12.22 | 3.98 | 15.43 | 13.26 | 12.71 | 8.11 | 9.79 |
|  | **Neutrophil count (10^3/μL)**  NVF 1.56-6.13; NVM 1.78-5.38 | 6.31 | 10.4 | 6.73 | 10.3 | 3.21 | 13.26 | 10.02 | 9.63 | 6.18 | 8.6 |
|  | **Lymphocyte count (10^3/μL)**  NVF 1.18-3.74; NVM 1.32-3.57 | 1.23 | 1.27 | 1.02 | 1.04 | 0.43 | 1.32 | 1.97 | 1.72 | 1.16 | 0.53 |
|  | **Neutrophils (%)**  NVF 34-71.1; NVM 34-67.9 | 77.8 | 82.9 | 80 | 84.3 | 80.6 | 85.9 | 17.5 | 75.8 | 76.3 | 88.7 |
|  | **Lymphocytes (%)**  NVF 19.3-51.7; NVM 21.8-53.1 | 15.1 | 10.5 | 12.1 | 8.5 | 10.8 | 8.6 | 14.9 | 13.5 | 14.3 | 5.4 |
|  | **Platelet count (10^3/μL)**  NVF 182-369; NVM 163-377 | 211 | 360 | 206 | 469 | 269 | 245 | 258 | 258 | 174 | 419 |
|  | **AST (U/L)** NVF 0-34; NVM 5-34 | 49 | 14 | 16 | 26 | 44 | 23 | 36 | 20 | 19 | 23 |
|  | **ALT (U/L)** NVF 10-49; NVM 0-55 | 23 | 10 | 7 | 18 | 21 | 7 | 19 | 18 | 16 | 18 |
|  | **GGT (U/L)** NVF 5-38; NVM 12-64 | - | 56 | 11 | 22 | 235 | 10 | 26 | 32 | 22 | 17 |
|  | **Urea (mg/dL)** NV 17-43 | 120 | 19 | 47 | 82 | 20 | 104 | 113 | 140 | 43 | 50 |
|  | **Creatinine (mg/dL)**  NVF 0.55-1.02; NVM 0.72-1.25 | 1.54 | 0.65 | 1.16 | 0.3 | 0.57 | 181 | 1.7 | 3.02 | 0.82 | 0.93 |
| **Post-operative day 1** | **Haematocrit (%)**  NVF 34.1-44.9; NVM 40.1-51.0 | 36.3 | 21.9 | 31 | 39.2 | 23.4 | 27.4 | 28.8 | 29.1 | 33 | 39.4 |
|  | **Haemoglobin (g/dL)**  NVF 11.2-15.7; NVM 13.7-17.5 | 11.7 | 6.9 | 10.3 | 12.4 | 7.8 | 9 | 9.5 | 9.3 | 10.7 | 12.2 |
|  | **White blood-cell count (10^3/μL)**  NVF 3.98-10.04; NVM 4.23-9.07 | 9.44 | 11.45 | 10.6 | 12.78 | 5.28 | 15.9 | 10.44 | 11.38 | 8.36 | 9 |
|  | **Neutrophil count (10^3/μL)**  NVF 1.56-6.13; NVM 1.78-5.38 | 8.43 | 9.12 | 9.15 | 10.92 | 4.24 | 13.27 | 6.79 | 7.77 | 6.95 | 8 |
|  | **Lymphocyte count (10^3/μL)**  NVF 1.18-3.74; NVM 1.32-3.57 | 0.81 | 1.59 | 0.89 | 1.05 | 0.6 | 1.2 | 2.47 | 2.09 | 0.59 | 0.43 |
|  | **Neutrophils (%)**  NVF 34-71.1; NVM 34-67.9 | 89.3 | 79.6 | 86.3 | 85.4 | 80.3 | 83.5 | 64.9 | 68.3 | 83.1 | 88.9 |
|  | **Lymphocytes (%)**  NVF 19.3-51.7; NVM 21.8-53.1 | 8.6 | 13.9 | 8.4 | 8.2 | 11.4 | 7.5 | 23.7 | 18.4 | 7.1 | 4.8 |
|  | **Platelet count (10^3/μL)**  NVF 182-369; NVM 163-377 | 199 | 354 | 158 | 345 | 320 | 129 | 232 | 235 | 171 | 350 |
|  | **AST (U/L)** NVF 0-34; NVM 5-34 | - | 26 | - | 56 | - | - | - | - | - | 45 |
|  | **ALT (U/L)** NVF 10-49; NVM 0-55 | - | 11 | - | 46 | - | - | - | - | - | 19 |
|  | **GGT (U/L)** NVF 5-38; NVM 12-64 | - | - | - | - | - | - | - | - | - | 19 |
|  | **Urea (mg/dL)** NV 17-43 | - | 37 | 85 | 78 | 37 | - | - | - | 30 | 49 |
|  | **Creatinine (mg/dL)**  NVF 0.55-1.02; NVM 0.72-1.25 | - | 0.63 | 1.14 | 0.61 | 0.64 | - | - | - | 0.64 | 1.11 |
| **Post-operative day 5 ± 2** | **Haematocrit (%)**  NVF 34.1-44.9; NVM 40.1-51.0 | 26.8 | 32.9 | 28.2 | 30.1 | 28.7 | 28.3 | 29.8 | 31.5 | 33.6 | 34.8 |
|  | **Haemoglobin (g/dL)**  NVF 11.2-15.7; NVM 13.7-17.5 | 8.4 | 11.2 | 9.4 | 9.9 | 9.4 | 8.2 | 9.6 | 10 | 11 | 10.7 |
|  | **White blood-cell count (10^3/μL)**  NVF 3.98-10.04; NVM 4.23-9.07 | 10.37 | 9.76 | 6.29 | 10 | 6.11 | 14.21 | 9 | 11.11 | 4.16 | 5.52 |
|  | **Neutrophil count (10^3/μL)**  NVF 1.56-6.13; NVM 1.78-5.38 | 7.86 | 7.16 | 5.45 | 7.79 | 4.98 | 11.42 | 5.77 | 8.14 | 2.09 | 4.19 |
|  | **Lymphocyte count (10^3/μL)**  NVF 1.18-3.74; NVM 1.32-3.57 | 1.85 | 1.89 | 0.71 | 1.4 | 0.77 | 1.33 | 2.11 | 1.74 | 1.19 | 0.62 |
|  | **Neutrophils (%)**  NVF 34-71.1; NVM 34-67.9 | 75.8 | 73.4 | 86.7 | 77.9 | 81.4 | 80.4 | 64.2 | 73.2 | 50.3 | 76 |
|  | **Lymphocytes (%)**  NVF 19.3-51.7; NVM 21.8-53.1 | 17.8 | 19.4 | 11.3 | 14 | 12.6 | 9.4 | 23.4 | 15.7 | 28.6 | 11.2 |
|  | **Platelet count (10^3/μL)**  NVF 182-369; NVM 163-377 | 138 | 408 | 272 | 227 | 375 | 168 | 264 | 352 | 245 | 325 |
|  | **AST (U/L)** NVF 0-34; NVM 5-34 | 27 | - | 25 | 40 | - | 34 | - | - | - | 38 |
|  | **ALT (U/L)** NVF 10-49; NVM 0-55 | 13 | - | 9 | 24 | - | 19 | - | - | - | 16 |
|  | **GGT (U/L)** NVF 5-38; NVM 12-64 | 12 | - | - | 18 | - | 39 | - | - | - | 26 |
|  | **Urea (mg/dL)** NV 17-43 | 153 | - | 37 | 55 | - | 157 | - | - | 19 | 49 |
|  | **Creatinine (mg/dL)**  NVF 0.55-1.02; NVM 0.72-1.25 | 1.46 | - | 0.85 | 0.47 | - | 1.35 | - | - | 0.54 | 0.74 |

**Abbreviations:** NV= normal values. NVF=normal values for females. NVM= normal values for males. AST=aspartate transaminase. ALT=alanine transaminase. GGT=gamma-glutamyltransferase
